# Evaluating Targeted Therapeutic Response With Predictive Blood-Based Biomarkers in Patients With Chronic Mild Traumatic Brain Injury

**DOI:** 10.1089/neur.2023.0003

**Published:** 2023-06-22

**Authors:** Shawn R. Eagle, Ava M. Puccio, Denes V. Agoston, Ryan Soose, Michael Mancinelli, Rachel Nwafo, Peyton McIntyre, Allison Agnone, Savannah Tollefson, Michael Collins, Anthony P. Kontos, Walter Schneider, David O. Okonkwo

**Affiliations:** ^1^ Department of Neurological Surgery, University of Pittsburgh, Pittsburgh, Pennsylvania, USA.; ^2^ School of Medicine, Uniformed Services University of the Health Sciences, Bethesda, Maryland, USA.; ^3^ Department of Otolaryngology, University of Pittsburgh, Pittsburgh, Pennsylvania, USA.; ^4^ Department of Orthopaedic Surgery, University of Pittsburgh, Pittsburgh, Pennsylvania, USA.; ^5^ Department of Psychology, University of Pittsburgh, Pittsburgh, Pennsylvania, USA.

**Keywords:** chronic mTBI, predictive biomarkers, p-tau, targeted treatment, UCH-L1

## Abstract

Chronic consequences of mild traumatic brain injury (mTBI) are heterogeneous, but may be treatable with targeted medical and rehabilitation interventions. A biological signature for the likelihood of response to therapy (i.e., “predictive” biomarkers) would empower personalized medicine post-mTBI. The purpose of this study was to correlate pre-intervention blood biomarker levels and the likelihood of response to targeted interventions for patients with chronic issues attributable to mTBI. Patients with chronic symptoms and/or disorders secondary to mTBI >3 months previous (104 days to 15 years; *n* = 74) were enrolled. Participants completed pre-intervention assessments of symptom burden, comprehensive clinical evaluation, and blood-based biomarker measurements. Multi-domain targeted interventions for specific symptoms and impairments across a 6-month treatment period were prescribed. Participants completed a follow-up testing after the treatment period. An all-possible model's backward logistic regression was built to identify predictors of improvement in relation to blood biomarker levels before intervention. The minimum clinically important difference (MCID) of the change score (post-intervention subtracted from pre-intervention) for the Post-Concussion Symptom Scale (PCSS) to identify treatment responders from non-responders was the primary outcome. The MCID for total PCSS score was 10. The model to predict change in PCSS score over the 6-month intervention was significant (*R*^2^ = 0.09; *p* = 0.01) and identified ubiquitin C-terminal hydrolase L1 (odds ratio [OR] = 2.53; 95% confidence interval [CI], 1.18–5.46; *p* = 0.02) and hyperphosphorylated tau (p-tau; OR = 0.70; 95% CI, 0.51–0.96; *p* = 0.03) as significant predictors of symptom improvement beyond the PCSS MCID. In this cohort of chronic TBI subjects, blood biomarkers before rehabilitation intervention predicted the likelihood of response to targeted therapy for chronic disorders post-TBI.

## Introduction

Approximately 3.2 million persons in the United States have disability secondary to mild (mTBI) traumatic brain injury (TBI).^1^ Chronic effects from mTBI are estimated to cost >$60 billion annually in the United States alone.^2^ Clinical manifestations of mTBI are heterogeneous, and modern management is predicated on the targeted treatment of specific conditions. Validation of objective assessments for the diagnosis and prognostication of mTBI has achieved recent success.^3,4^ Focus is expanding to investigate additional contexts of use for blood biomarkers or clinical characteristics to predict the therapeutic response to a targeted intervention for mTBI. The Centers for Disease Control and Prevention refers to this class of biomarkers as “predictive biomarkers.”^5^ The purpose of this exploratory study was to assess the relationship between pre-intervention blood biomarker concentrations and clinical characteristics with the magnitude of symptom improvement after a 6-month targeted intervention for chronic symptoms related to mTBI.

## Methods

### Design and participants

The TEAM-TBI (Targeted Evaluation Action and Monitoring of TBI) study was a prospective multiple interventional trial of patients with sequelae secondary to mTBI >3 months previous (NCT02657135). Inclusion criteria were 18–60 years of age and fluent in the English language with a documented history of TBI or blast exposure since 2001. Interventions were targeted to the patient's impairments over 6 months. Clinical results of this trial have been published.^6^ Results from earlier work focused on symptoms and clinical grading scales whereas the present analysis focused on measured biomarkers.

Pre- and post-intervention assessments were conducted, including demographics/medical history (e.g., sex, injury mechanism, number of mTBIs, time since index injury, and military status), the Post-Concussion Symptom Scale (PCSS), and blinded analysis of blood biomarkers (e.g., glial fibrillary acidic protein [GFAP], tau, hyperphosphorylated tau Thr231 [p-tau], von Willebrand factor [vWF], brain lipid binding protein [BLBP], ubiquitin C-terminal hydrolase-L1 [UCH-L1], vascular endothelial growth factor-a [VEGFa], and claudin-5 [CLDN5]). These biomarkers were chosen before study initiation for analysis, based on their empirical association with mTBI and/or common mTBI sequelae. This study was approved by the University of Pittsburgh Institutional Review Board for human subjects' research.

### Blood biomarker analysis

Plasma samples were analyzed by using the reverse phase protein microarray (RPPM) system, a high-sensitivity, high-throughput proteomics platform according to established procedures.^7–16^ Denatured samples were serially diluted in a 1:2 manner (five-step) and printed onto nitrocellulose film slides by using a Quanterix 2470 Arrayer (Quanterix, Billerica, MA). Slides were dried and blocked with Azure Protein-Free Blocking Buffer (Azure Biosystems, Inc, Dublin, CA) and incubated with primary antibodies overnight (8–12 h) at 4°C. A list of primary antibodies can be viewed in Supplementary Table S1. After washing, slides were incubated with biotinylated secondary antibodies (1:100,000 dilution). Slides were scanned in an Innopsys InnoScan 710-IR scanner for XDR (extended dynamic range) signal acquisition at 785 nm (Innopsys Inc, Chicago, IL). The reverse phase protein array (RPPA) or RPPM, a high-sensitivity, high-throughput antibody-based analytical platform, was adopted from MD Anderson Cancer Center's protocol.^17^ We have successfully used RPPA in numerous TBI-related projects during the last decade. After incubation with the primary antibodies (see Supplementary Table S1 for details), slides were washed, blocked, and incubated with fluorescent-tagged secondary anti-mouse or -rabbit antibodies (from Invitrogen) diluted at 1:6000 in antibody incubation buffer for 1 h at room temperature.

Fluorescence data were imported into a Microsoft Excel-based bioinformatics program (Microsoft Corporation, Redmond, CA), and net intensity versus dilution was plotted on a log2-log2 scale. Total amount of antigen was determined by the y-axis intercept or Y-cept (i.e., by extrapolating the regression line to zero). Here, we express the Y-cept values as log2-transformed Y-cept values, which correspond to the total net intensity of the undiluted plasma sample.

### Outcome measures

#### Post-Concussion Symptom Scale

The PCSS is a 22-item survey completed on a 7-point Likert scale, from 0 (not at all) to 6 (severe). PCSS score was the primary outcome measure for the present study.

### Statistical analysis

Pre- and post-intervention PCSS scores were converted into change scores (post-intervention score – pre-intervention score). The MCID of PCSS change score was determined by multiplying 0.5 (signifying a medium Cohen's *d* effect size) with the standard deviation of the total sample. Univariable logistic regressions were conducted to assess the association of individual biomarkers with improving PCSS score beyond the MCID. An all-possible backward logistic regression model was built to assess the association of pre-intervention blood biomarker concentrations and demographics/medical history variables on improving outcome scores (cutoff, *p* < 0.05). Odds ratios (ORs) and 95% confidence intervals (CIs) were derived from the models. For interpretation, ORs >1 are indicative of higher odds of improving greater than the MCID, whereas ORs <1 are indicative of lower odds of improving greater than the MCID. *Post hoc* diagnostics were conducted to evaluate model performance. Internal validity testing was conducted using the *calibrationbelt* command in Stata software (SE 17.0; StataCorp LLC, College Station, TX). A *p* value <0.05 indicates that the model was poorly calibrated. Predicted probabilities of the model were generated to calculate discriminative validity using receiver operating characteristic area under the curve (AUC) analysis and the Brier score to calculate the accuracy of probabilistic predictions. A Brier score of 0 indicates perfect accuracy, and 1 indicates perfect inaccuracy.

## Results

### Demographics

Participants (*n* = 74; age: 35.8 ± 8.6 years; 19% female) were 5.6 ± 3.6 years from their index mTBI and reported a median of five mTBI exposures at enrollment (Table 1). The sample was 67.4% former military (*n* = 64). Reported mechanisms of injury were blunt (*n* = 35; 36.8%), blast (*n* = 24; 25.3%) or both (*n* = 36; 37.8%).

**Table 1.** Demographic and Clinical Outcome Variables for the Sample

	Mean ± SD No. (%)	PCSS^+^* n* = 50	PCSS^–^* n* = 34	*p* value
Age at enrollment	35.8 ± 8.6	34.3 ± 8.0	38.7 ± 9.0	0.02^*^
Years since last TBI	5.6 ± 3.6	5.72 ± 35.00	5.02 ± 3.70	0.38
Mechanism of index TBI Blast Blunt Both	24 (24.7) 35 (36.1) 36 (37.1)	15 (30.0) 18 (36.0) 17 (34.0)	6 (17.6) 13 (38.2) 15 (44.1)	0.66 0.84 0.35
Female sex	18 (18.6)	10 (20.0)	6 (17.6)	0.79
Military status	64 (67.4)	36 (72.0)	22 (64.7)	0.48
Exposures	5.2 ± 3.5	5.1 ± 3.2	5.7 ± 3.9	0.53
Pre-intervention PCSS total	49.3 ± 22.9	55.4 ± 21.6	40.1 ± 22.1	0.002^*^
Post-intervention PCSS total	35.4 ± 22.6	28.5 ± 21.0	45.5 ± 21.3	<0.001^*^
Change in PCSS total	–13.8 ± 20.3	–26.9 ± 13.7	5.4 ± 10.7	<0.001^*^

^*^ Indicates statistically significant at *p* < 0.05.

TBI, traumatic brain injury; PCSS, Post-Concussion Symptom Scale; SD, standard deviation.

The MCID for PCSS change score was −10. Participants whose symptoms improved by more than −10 (PCSS^+^; *n* = 50) were younger at enrollment (4.3 years), with higher pre-intervention PCSS total scores (+15.3) and lower post-intervention total scores (−17.0) than participants whose symptoms improved by −10 or less (PCSS^–^; *n* = 34).

### Univariable regression models

Logistic regression models to differentiate PCSS^+^ from PCSS^–^ with individual biomarkers can be viewed in Table 2. No biomarkers had a statistically significant relationship with symptom improvement beyond the MCID, but UCH-L1 had the highest OR (OR = 1.76; 95% CI, 0.91–3.40; *p* = 0.09).

**Table 2.** Univariable Logistic Regression Results to Predict Concussion Symptom Improvement After Targeted Intervention Using Individual Blood Biomarkers

	Odds ratio	*p *value	95% CI
UCHL1	1.76	0.09	0.91–3.40
GFAP	1.15	0.67	0.61–2.14
vWF	1.03	0.90	0.68–1.56
VEGFa	1.19	0.51	0.71–2.01
p-tau	0.82	0.15	0.63–1.07
BLBP	1.61	0.16	0.83–3.13
CLDN5	1.57	0.17	0.82–3.03
Tau	1.18	0.48	0.74–1.89

UCHL1, ubiquitin C-terminal hydrolase L1; GFAP, glial fibrillary acidic protein; vWF, von Willebrand factor; VEGFa, vascular endothelial growth factor-a; p-tau, hyperphosphorylated tau; BLBP, brain lipid binding protein; CLDN5, claudin-5; CI, confidence interval.

### Multi-variable regression model

The model to differentiate PCSS^+^ from PCSS^–^ over the 6-month intervention period was statistically significant (*R*^2^ = 0.09; *p* = 0.01) and retained two predictors (Table 3). UCH-L1 (OR = 2.54; 95% CI, 1.18–5.46; *p* = 0.017) was associated with higher odds of symptom improvement beyond the MCID, whereas p-tau (OR = 0.70; 95% CI, 0.51–0.96; p = 0.026) was associated with lower odds of symptom improvement beyond the MCID. *Post hoc* internal validity testing indicated that the model was well calibrated (*p* = 0.35). The final model had acceptable discriminative validity (AUC = 0.69; 95% CI, 0.56–0.82; *p* = 0.006), with a sensitivity of 77.3% and specificity of 50%. The Brier score was 0.21, indicating reasonable forecasting ability. No clinical characteristics were significant predictors.

**Table 3.** Multi-Variable Logistic Regression to Predict Concussion Symptom Improvement After Targeted Intervention

	Odds ratio	*p *value	95% CI
UCH-L1	2.54	0.017	1.18–5.46
p-tau	0.70	0.026	0.51–0.96

UCH-L1, ubiquitin C-terminal hydrolase L1; p-tau, hyperphosphorylated tau; CI, confidence interval.

## Discussion

This study identified predictive biomarkers for both a positive and negative response to targeted intervention in chronic mTBI. Higher pre-intervention UCH-L1 was associated with 154% higher odds of improving global concussion symptoms (i.e., PCSS scores) beyond the MCID. Conversely, higher pre-intervention p-tau was negatively associated with 30% lower odds of symptom improvement beyond the MCID after targeted intervention. These preliminary results for predictive biomarkers of targeted intervention for chronic TBI require further study and validation in larger mTBI populations.

UCH-L1 is an enzyme found in nerve cells throughout the brain involved in the clearance of unneeded proteins.^18^ Blood level of UCH-L1 is elevated after subconcussive blast exposure during military training and can remain elevated for years after moderate and severe TBI. UCH-L1 is a potential marker of treatment efficacy in patients with chronic neurological injury. Perhaps, higher levels of UCH-L1 have the capacity to improve symptoms and function after mTBI or subconcussive exposure by removing cortical proteins associated with these exposures and poorer clinical outcomes (e.g., amyloid beta, tau, and p-tau). This hypothesis may explain why UCH-L1 and p-tau were not significantly associated with symptom improvement as univariate predictors, but were both significantly associated with symptom improvement when considered together. Indeed, impairment of the ubiquitin-proteasome pathway (which can occur after TBI)^19^ has been shown to enhance the toxic accumulation of proteins (such as p-tau) and is associated with neurodegenerative disease, such as Parkinson's and Alzheimer's diseases.^20,21^

p-tau is the hyperphosphorylated tau protein typically associated with neurofibrillary tangles in Alzheimer's disease.^22^ p-tau detected in the blood can differentiate military veterans with an mTBI history from those with no history, and p-tau correlates significantly with post-concussive symptom burdens.^23^ p-tau formation is associated with synaptic dysfunction and neuronal loss, both mechanisms of memory impairments.^22^ Based upon this evidence and the results of this preliminary study, higher levels of p-tau in the blood of chronic mTBI patients may reflect a neuronal environment not conducive to improvement from non-pharmacological treatment.^24^

Although this seems a plausible hypothesis for these preliminary results, it should be noted that PCSS scores were 15 points higher before intervention and ∼17 points lower post-intervention in the group of responders compared to non-responders. This could represent a regression to the mean for the responder group who had much higher symptom burdens before treatment. Non-responders were roughly 4.5 years older than responders (Table 1), which may have impacted the degree of improvement, but age was not retained in the final regression model as a significant predictor. In the absence of other group differences which may have impacted the outcome (i.e., years since last TBI, proportion of females and veterans, and exposures), it is unclear why responders had higher symptom burdens before intervention.

### Limitations

The final model only accounted for 9% of variance in PCSS improvement from targeted intervention, indicating that other predictors are necessary to understand who will improve from targeted treatment in this unique population of patients with chronic issues related to mTBI. The sample was approximately two thirds former military, and >50% of the index injuries were related to a blast mechanism. This limits the generalizability of these results to the general population or athlete cohorts who are typically not exposed to blast mechanisms or military training. Many of the participants had missing data for follow-up (*n* = 21; 22%), resulting in a potential bias of PCSS change scores, so only participants with complete data were retained for analysis in the present study. Adherence to the prescribed therapies was not reliably available for this analysis and could help explain differences between groups. The wide range of time since last mTBI for the included participants may have introduced a temporal variance, which we were unable to account for because of the smaller sample size.

## Conclusion

In this secondary analysis of a prospective interventional trial in mTBI, higher pre-intervention levels of UCH-L1 served as a predictive biomarker of greater capacity to improve from targeted intervention for chronic issues related to mTBI. Higher pre-intervention levels of p-tau may reflect a biomolecular environment resistant to symptom improvement from chronic mTBI-related symptoms. Future research is needed to confirm and extend this finding in larger cohorts with generalizable populations, given that UCH-L1 and p-tau accounted for ∼10% of the variance in symptom improvement from a targeted intervention in a small cohort of participants with chronic issues from mTBI.

## Supplementary Material

Supplemental data

## Abbreviations Used

AUC: area under the curve
BLBP: brain lipid binding protein
CI: confidence interval
CLDN5: claudin-5
GFAP: glial fibrillary acidic protein
MCID: minimum clinically important difference
mTBI: mild TBI
OR: odds ratio
PCSS: Post-Concussion Symptom Scale
p-tau: hyperphosphorylated tau
RPPA: reverse phase protein array
RPPM: reverse phase protein microarray
TBI: traumatic brain injury
UCH-L1: ubiquitin C-terminal hydrolase L1
VEGFa: vascular endothelial growth factor-a
vWF: von Willebrand factor
