# Supplementary material for: Evaluating Targeted Therapeutic Response With Predictive Blood-Based Biomarkers in Patients With Chronic Mild Traumatic Brain Injury
Source: Neurotrauma Rep. 2023 Jun 22;4(1):404–9. doi: 10.1089/neur.2023.0003 (PMC10288300; doi:10.1089/neur.2023.0003)
Supplement: Supplemental data [file Suppl_TableS1.docx]

| **Supplemental Table 1. List of primary antibodies for included biomarkers** | | | |
| --- | --- | --- | --- |
| **Name of protein** | **Manufacture** | **Catalog #** | **Immunogen/Clonality** |
| UCHL-1 | Cell Signaling | 11896 | Rabbit Monoclonal |
| Tau | Cell Signaling | 4019 | Mouse Monoclonal |
| p-Tau | EMD Millipore | SAB4504563 | Rabbit Polyclonal |
| GFAP | Abcam | ab7260 | Rabbit Polyclonal |
| BLBP | EMD Millipore | ABN14 | Rabbit Polyclonal |
| CLDN5 | EMD Millipore | ABT45 | Rabbit Polyclonal |
| VEGF-A | Abcam | ab53465 | Rabbit Polyclonal |
| vWF | Abcam | ab181871 | Rabbit Monoclonal |
